# Positron Emission Tomography with [^18^F]FLT Revealed Sevoflurane-Induced Inhibition of Neural Progenitor Cell Expansion *in vivo*

**DOI:** 10.3389/fneur.2014.00234

**Published:** 2014-11-17

**Authors:** Shuliang Liu, Merle G. Paule, Xuan Zhang, Glenn D. Newport, Tucker A. Patterson, Scott M. Apana, Marc S. Berridge, Mackean P. Maisha, William Slikker, Cheng Wang

**Affiliations:** ^1^Division of Neurotoxicology, National Center for Toxicological Research, U.S. Food and Drug Administration, Jefferson, AR, USA; ^2^3D Imaging, LLC, Little Rock, AR, USA; ^3^Division of Bioinformatics and Biostatistics, National Center for Toxicological Research, U.S. Food and Drug Administration, Jefferson, AR, USA

**Keywords:** neural progenitor cell, positron emission tomography, [^18^F]FLT, sevoflurane, proliferation

## Abstract

Neural progenitor cell expansion is critical for normal brain development and an appropriate response to injury. During the brain growth spurt, exposures to general anesthetics, which either block the *N*-methyl-d-aspartate receptor or enhance the γ-aminobutyric acid receptor type A can disturb neuronal transduction. This effect can be detrimental to brain development. Until now, the effects of anesthetic exposure on neural progenitor cell expansion *in vivo* had seldom been reported. Here, minimally invasive micro positron emission tomography (microPET) coupled with 3′-deoxy-3′ [^18^F] fluoro-l-thymidine ([^18^F]FLT) was utilized to assess the effects of sevoflurane exposure on neural progenitor cell proliferation. FLT, a thymidine analog, is taken up by proliferating cells and phosphorylated in the cytoplasm, leading to its intracellular trapping. Intracellular retention of [^18^F]FLT, thus, represents an observable *in vivo* marker of cell proliferation. Here, postnatal day 7 rats (*n* = 11/group) were exposed to 2.5% sevoflurane or room air for 9 h. For up to 2 weeks following the exposure, standard uptake values (SUVs) for [^18^F]-FLT in the hippocampal formation were significantly attenuated in the sevoflurane-exposed rats (*p* < 0.0001), suggesting decreased uptake and retention of [^18^F]FLT (decreased proliferation) in these regions. Four weeks following exposure, SUVs for [^18^F]FLT were comparable in the sevoflurane-exposed rats and in controls. Co-administration of 7-nitroindazole (30 mg/kg, *n* = 5), a selective inhibitor of neuronal nitric oxide synthase, significantly attenuated the SUVs for [^18^F]FLT in both the air-exposed (*p* = 0.00006) and sevoflurane-exposed rats (*p* = 0.0427) in the first week following the exposure. These findings suggested that microPET in couple with [^18^F]FLT as cell proliferation marker could be used as a non-invasive modality to monitor the sevoflurane-induced inhibition of neural progenitor cell proliferation *in vivo*.

## Introduction

Animals exposed neonatally to general anesthetics develop cognitive deficits and behavioral abnormalities later in their lives (1–3). And accumulating preclinical evidence indicates the toxic effect of general anesthesia to developing brain (4–12). Therefore, substantial concerns over the safety of using general anesthetics in obstetric and pediatric patients have arisen (13–16). The pathogenesis of anesthetic neurotoxicity to developing central nervous system (CNS) has been under extensive investigation (4–7, 17–20).

In the developing CNS, the excitatory signal conferred by γ-aminobutyric acid and glutamate via synaptic transmission is neural trophic in nature to neurons growth and critical to orchestrate the neurons to form neuronal circuits (21–23). The γ-aminobutyric acid type A receptors (GABA_A_Rs) and *N*-methyl-d-aspartate receptors (NMDARs) are the primary receptors, which mediated the excitatory signal transmission. In anesthesia and sedations, the common anesthetics are believed to act either as the agonist to GABA_A_Rs, or as the antagonist to NMDARs (24–26). Sevoflurane [fluoromethyl 2,2,2-trifluoro-1-(trifluoromethyl) ethyl ether], for example, a volatile anesthetic commonly used in anesthesia and sedations in pediatric patients, is considered to act as an agonist to the GABA_A_Rs at anesthesia relevant concentration (27). Lengthy exposure of immature CNS to anesthetic agents that target on these receptors would conceivably affect the CNS development adversely.

It has been agreed that the animals during the brain growth spurt period are most susceptible to the anesthetics, when the formations of synapses are at the peak level (28). Prolonged exposure of animals in brain growth spurt period to general anesthetics results in massive neuronal apoptosis in the cerebral cortex (1, 5). In addition, following neonatal exposure to anesthetic, the number of synapses and dendritic spine density are altered in cerebral cortex, including the hippocampal formation (7, 29). And the attenuating effect of anesthetic exposure on dendritic spine could extend into the adulthood of the rats, likely to contribute to the abnormal behavior and cognitive impairment.

Furthermore, the anesthetic-induced compromised memory and learning performance may also be associated with the inhibition of neurogenesis in the hippocampus. The proliferation of neurons and astrocytes from pleural-potent progenitor cells is an ongoing process after birth. The dentate gyrus (DG) of hippocampal formation and the subventricular zone (SVZ) are the two discrete brain regions where neurogenesis continued after birth (30, 31). Therefore, the anesthetic-induced behavioral abnormalities and cognitive deficits found later in the life could be related to the compromised neurogenesis (9). Data are accumulating that support the hypothesis that common anesthetics, which activate the GABA_A_R exert inhibitory effect on the progenitor cells in hippocampus (32–38). And the anesthetic-induced insult on neurogenesis is more severe when the exposure was imposed in the early postnatal period than in the adulthood (9, 32).

In most of the reported *in vivo* studies on the effect of anesthetic exposure to neurogenesis, the duration of the anesthetic-induced inhibition on neurogenesis remained to be investigated. This was due to the fact that the animals were sacrificed on the time points of study. In contrast, longitudinal study using non-invasive methods of examination would allow the evaluation the time course of anesthetic-induced pathologies and to monitor the effects of treatment. Therefore, it is justified to search for minimally invasive approaches that could be repeated in the same subjects at different times. Furthermore, it is necessary to search for and test the minimally invasive approaches in translational studies before they could be used in clinical settings to provide evidence of neurotoxicity directly.

In our studies, micro positron emission tomography (microPET) is utilized to depict the neuronal apoptosis and inflammatory alterations following neonatal exposure to anesthetics *in vivo* (39–41). A minimally invasive imaging modality, PET coupled with radiolabeled tracer is used to analyze specifically targeted biological event in the subject (42). microPET has the high sensitivity and high-spatial-resolution required in the imaging of small animals (43, 44). Coupled with radioactively labeled ligands, it has been used to assess the binding of the ligand by the tissue interested quantitatively or semi-quantitatively.

**Figure 1:**
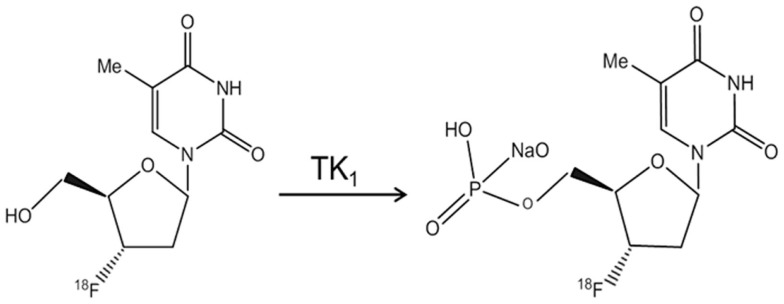
**The structure of [^18^F]FLT and its phosphorylation by thymidine kinase 1 (TK_1_)**.

The effect of sevoflurane exposure on the proliferation of neural progenitor cells has not been evaluated longitudinally with microPET so far. To measure the cell proliferation of endogenous origin, in the current study, we propose to use [^18^F]3′-deoxy-3′-fluoro-l-thymidine ([^18^F]FLT) as the cell proliferation tracer (45, 46). FLT is an analog of thymidine and taken up by cells and phosphorylated by thymidine kinase 1 (TK_1_), leading to the intracellular trapping within the cells without being incorporated into the cellular DNA (Figure 1). The activity of TK_1_ was closely regulated in conjunction with the cell proliferation. Thus, [^18^F]FLT has been used as PET radiotracer to assess the DNA synthesis through salvage pathway in a quantitative measure (47).

In addition, we also seek for a pharmaceutical agent to counteract the effect of sevoflurane on the neural cell expansion. 7-nitroindazole (7-NI) is a selective inhibitor of neural nitric oxide synthase (nNOS). It was demonstrated to be protective against the ketamine-induced neuronal death in rat forebrain culture (48). And inhibition of nitric oxide synthase (NOS) by NOS inhibitor had been shown to significantly increase the population of proliferating cells in SVZ (31). Therefore, in this study, we assessed the effect of sevoflurane exposure on cell proliferation following the sevoflurane exposure on postnatal day (PND) 7 with microPET in conjunction with [^18^F]FLT over a period of 4 weeks. Meanwhile, the role of NOS inhibition with 7-NI in neural progenitor cell proliferation was evaluated. It was demonstrated that non-invasive imaging with microPET using [^18^F]FLT detected the inhibition of neural progenitor cell proliferation in the first 2 weeks following the sevoflurane exposure and revealed the attenuating effects of 7-NI to neural progenitor cell expansion.

## Materials and Methods

### Animals

The Institutional Animal Care and Use Committee (IACUC) at the National Center for Toxicological Research (NCTR) approved the experimental protocol. And all animal procedures were conducted in full accordance with the public health service (PHS) Policy on Humane Care and Use of Laboratory Animals.

Male and female Sprague–Dawley neonatal rats born from the NCTR breeding colony were used. All rat pups were maintained with their dam in the animal facility at NCTR (8–10 pups/dam/cage) with room temperature maintained at 22 ± 2°C. The animals were provided *ad libitum* standard rat chow and tap water under a light/dark cycle of 12/12 h, where the light cycle began at 6:00 a.m. Rat pups of the same gender were weaned into one cage (2–3 rats/cage) at 3 weeks of age. A total of 32 rats were randomly assigned to experimental groups as follows: sevoflurane treated (*n* = 11), sevoflurane treated with 7-NI (*n* = 5); control (*n* = 11), and controls treated with 7-NI (*n* = 5). The animals were from four litters in total. They have been randomly grouped into four groups. Each group of neonatal rats was consisted of those from different litters.

### Neonatal exposure to sevoflurane

Neonatal rats weighted around 15–20 g on PND 7 were exposed to sevoflurane within a clear anesthesia induction chamber (E-Z Anesthesia^®^, Palmer, PA, USA). Sevoflurane (Webster Veterinary Supply, Sterling, MA, USA) along with oxygen was delivered from a sevoflurane specific vaporizer (Tec 7, Baxter, Dallas, TX, USA) at the concentration of 2.5% (v/v) into the chamber at a rate of about 0.5–1 l/min. The induction chamber was warmed from the bottom using a water heated pad set at 40°C. A charcoal filter canister was used to absorb the extra vaporized anesthetic from the chamber. The body temperature and blood oxygen saturation levels of anesthetized animals were monitored every hour via pulse oximetery (Mouse OX Plus, STARR Life Sciences, Oakmont, PA, USA). Sevoflurane exposures lasted 9 h, after which the rat pups were returned to their dams. Control rat pups remained with their dam and received room air. 7-NI (Sigma-Aldrich) was dissolved in corn oil and sterile filtered (0.45 μm), was administrated via *i.p*. injection at the dosage of 30 mg/kg body weight at 18 and 1 h prior to and 4 h following the start of the 9 h-exposure to either sevoflurane or room air. All rats were returned to and kept in the animal facility for subsequent microPET studies.

### Preparation of [^18^F]FLT

[^18^F]3′-deoxy-3′-fluoro-l-thymidine was prepared by 3D Imaging LLC (Little Rock, AR, USA) following procedures published previously (49, 50). Briefly, [^18^F]fluoride was reacted with 5′-*O*-(Benzoyl)-2,3′-anhydrothymidine. Deprotection with sodium hydroxide afforded crude [^18^F]FLT. The reaction mixture was diluted with sodium phosphate and passed through an alumina cartridge. Final purification was accomplished by reverse phase HPLC eluted with 10% ethanol in saline. The collected product was sterile filtered for i.v. administration. Typically, about 7.5 GBq (200 mCi) of [^18^F]FLT was produced at the end of the synthesis, 60 min EOB, from 75 GBq (2 Ci) of fluoride. The FLT had specific activity at 60 min EOB of 1.1–2.2 TBq (30–60 Ci) per micromole.

### microPET image acquisition

All images of the rat brain were acquired quantitatively utilizing a Focus 220, high-resolution small animal PET scanner (Siemens Preclinical Solution, Knoxville, TN, USA). The scanner has 96 lutetium oxyortho-silicate (LOS) detectors and provides a transaxial resolution of 1.35 mm full-width at half-maximum (FWHM) at the center of field of view. Data were collected in a 128 × 128 × 95 matrix with a pixel width of 0.475 mm and a slice thickness of 0.815 mm.

For all microPET scans, animals were induced and maintained under anesthesia with isoflurane (1.5%) blended with oxygen and delivered via a homemade face mask. [^18^F]FLT was administrated on PNDs 14, 21, and 35 (18.5 MBq/dose, *i.p*.). microPET scans were performed for 90 min, beginning 30 min after *i.p*. injection of the radiolabeled FLT. A set of three dimensional microPET images (1 frame every 5 min, 18 frames) was reconstructed over the 90 min scanning period.

### microPET data analysis

Medical image analysis software, ASIPro™(Concorde Microsystems, Inc., Knoxville, TN, USA) was used in the quantitative analyses of the imaging data. Hippocampus was supposed to be one of two sites in the brain where neurogenesis continue after birth. Therefore, bilateral hippocampus regions were selected as the locations for the regions of interest (ROIs). Three dimensional ROIs, 5 pixels in diameter were drawn in the coronal plane with reference to transverse and sagittal planes displayed simultaneously. The radioactivity following [^18^F]FLT injection was measured using the software provided by ASIPro™. [^18^F]FLT accumulation in the ROIs was converted into standard uptake values [SUVs = average concentration of radioactivity in ROI (mCi/ml)/injected dose (mCi)/body weight (g)].

### Statistical analysis

Statistical analysis system (SAS) for Windows (v9.3) was used in the data analysis. SigmaPlot for windows (v11.0) was used in generating graphs. The averaged value of bilateral hippocampal region SUVs collected from each frame in the scan was used in the statistical analysis. The average SUVs of groups are presented as mean ± SEM. Since the data in each subject were acquired by repeated measures, the linear mixed effect model was utilized in the analysis. The effects of treatments (air versus sevoflurane exposure), 7-NI co-administration, and time interval between the exposure and microPET scan (1 week versus 2 or 4 weeks) were examined in the analysis. Dunnett’s multiplicity adjustment test was used for comparisons with the control in order to preserve the overall type I error rate at the nominal 5% level. Statistical significance was considered when the *p*-value is <0.05.

## Results

F LT uptake in hippocampal region following sevoflurane exposure at PND 7 revealed by serial microPET scans]

### Attenuated [^18^F]FLT uptake in hippocampal region following sevoflurane exposure at PND 7 revealed by serial microPET scans

**Figure 2:**
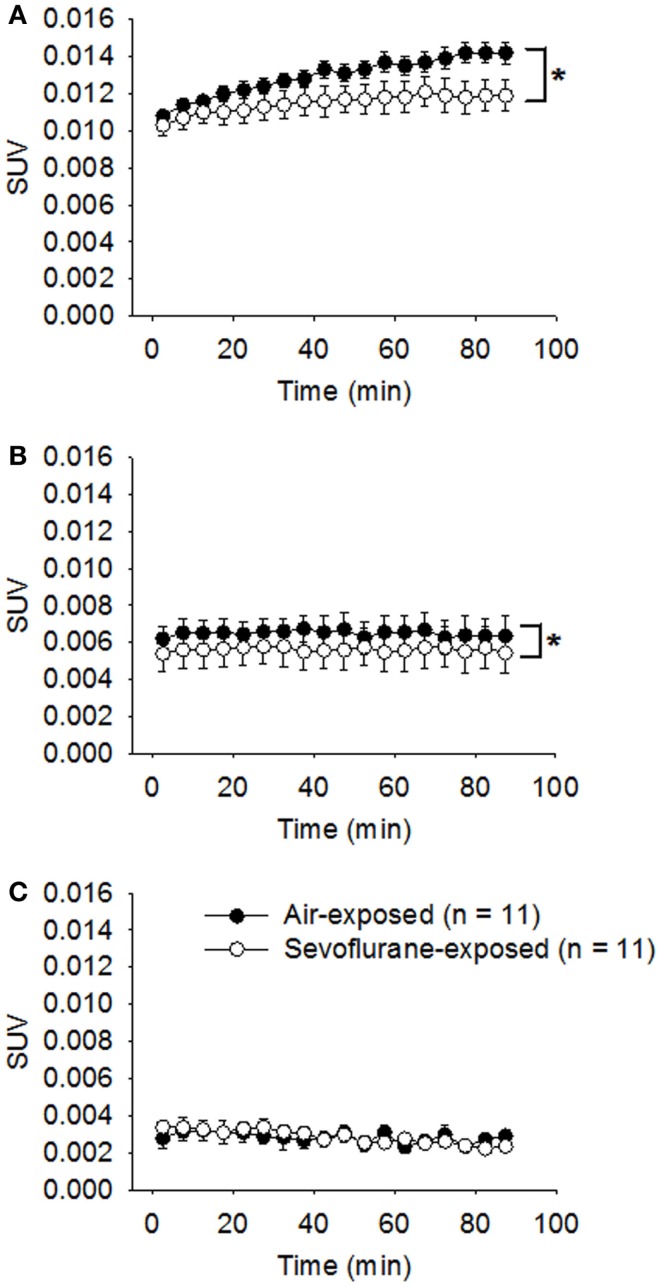
**Attenuated [^18^F]FLT uptake in the hippocampal region was revealed by serial microPET scans for the first 2 weeks following sevoflurane exposure on PND 7**. On PND 7, the treated rats were exposed to sevoflurane (2.5%) mixed with oxygen for 9 h (*n* = 11); the control rats (*n* = 11) were exposed to room air. One **(A)**, 2 **(B)**, and 4 **(C)** weeks following the exposure, the animals were examined using microPET (scan time = 90 min) following the administration of [^18^F]FLT (18.5 MBq/dose) by *i.p*. injection. Standard uptake values (SUVs) are presented as means ± SEM. The [^18^F]FLT uptake in the hippocampus of sevoflurane-exposed rats was significantly attenuated compared with air-exposed rats in weeks 1 (*p* < 0.0001) and 2 (*p* = 0.0108) following the anesthetic exposure. (*p < 0.05, repeated measures linear mixed effect model). By week 4, the SUVs in the sevoflurane-exposed rats and the air-exposed rats were no longer significantly different (*p* = 0.9047, repeated measures linear mixed effect model).

All the neonatal rats survived the 9-h exposure of 2.5% sevoflurane without disturbed respiration or cyanosis on PND 7. The developing animals were examined with microPET scans in conjunction with [^18^F]FLT administration at 1, 2, and 4 weeks following the exposure. The statistical analysis on the sevoflurane-exposed rats and control rats showed that both the sevoflurane exposure (*p* = 0.0002) and the timing of microPET scanning (*p* < 0.0001) affected the SUVs of hippocampal regions significantly, and the effect of sevoflurane exposure on SUVs was significantly dependent on that time interval between the exposure and PET scan (*p* = 0.0012). In week 1 following the exposure (PND 14), the average SUV of sevoflurane-exposed rats (0.0115 ± 0.000176, *n* = 11) was significantly (*p* < 0.0001) lower in comparison with that of control rats (0.0130 ± 0.000129, *n* = 11) (Figure 2A). The significantly lower [^18^F]FLT uptake and retention in sevoflurane-exposed rats could suggest that the cell proliferation in the hippocampal regions was inhibited in the first week following the 9-h sevoflurane exposure. Subsequently, in week 2 (PND 21), the difference in SUVs between the sevoflurane-exposed group (0.00578 ± 0.000198) and the control group (0.00649 ± 0.000186) remained significant (*p* = 0.0108; Figure 2B). By week 4 (PND 35), the SUVs in the sevoflurane-exposed rats (0.00286 ± 0.0000766) and in the control group (0.00283 ± 0.000101) were similar (*p* = 0.9047; Figure 2C).

### Administration of 7-NI inhibited [^18^F]FLT uptake in the hippocampal region

**Figure 3:**
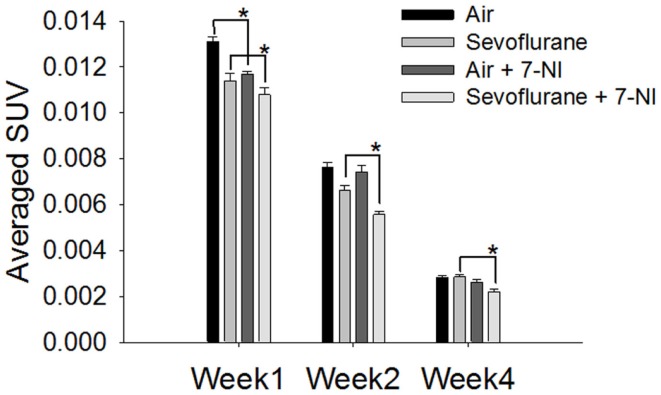
**Co-administration of 7-NI induced inhibition upon [^18^F]FLT uptake in the hippocampal regions in either air- or sevoflurane- exposed rats**. On PND 7, the rats (*n* = 5) were treated with 7-NI *i.p*. (30 mg/kg body weight, dissolved in corn oil) 18 and 1 h prior to and 4 h following the beginning of 9 h-exposure to either sevoflurane or air. One, 2, and 4 weeks after exposure the animals were assessed via microPET 30 min after the injection of [^18^F]FLT *i.p*. (18.5 MBq/dose). In week 1, the [^18^F]FLT uptake of the hippocampus region in rats co-administrated with 7-NI was significantly lower than those in rats exposed to either air (*p* = 0.00006) or sevoflurane (*p* = 0.0427) only. In week 4 after exposure, the SUV in sevoflurane-exposed plus 7-NI group remained significantly lower than that of group exposed to sevoflurane only (*p* = 0.0077, Dunnett’s test). SUVs are presented as means ± SEM, asterisks denote significant differences in [^18^F]FLT uptake between the rats co-administrated 7-NI and those exposed to air or sevoflurane alone in weeks 1, 2 or 4 after exposure (*p < 0.05, repeated measures linear mixed effect model, Dunnett’s test for multiple comparisons).

7-Nitroindazole was co-administered to rats exposed either to sevoflurane or air to test if it could neutralize the effect of sevoflurane exposure on the neural progenitor cell proliferation. The effects of 7-NI co-administration on the SUVs were significant (*p* < 0.0001) as evaluated with the mixed effect model. In week 1 following the exposure, the [^18^F]FLT uptake in the hippocampal regions in rats exposed to air with co-administrated 7-NI (0.0117 ± 0.000117, *n* = 5) was reduced significantly compared with those in the rats exposed to air alone (0.0131 ± 0.0002, *n* = 5; *p* = 0.00006, Dunnett’s test, Figure 3); the co-administration of 7-NI in the rats exposed to sevoflurane further attenuated the SUVs of the hippocampal regions in comparison with those in the rats exposed to sevoflurane alone (*p* = 0.0427, Dunnett’s test; Figure 3). In week 2, the SUVs of the air-exposed group were similar to those of the air-exposed with 7-NI co-administration (*p* = 0.8701, Dunnett’s test). In contrast, the SUVs of the rats exposed to sevoflurane plus 7-NI co-administration (0.00558 ± 0.000117) remained significantly (*p* = 0.0043) lower than those of the rats exposed to sevoflurane only (0.00663 ± 0.000222). In week 4, the SUV in sevoflurane-exposed plus 7-NI co-administration group (0.00222 ± 0.000128) remained significantly (*p* = 0.0077, Dunnett’s test) lower than that of the group exposed to sevoflurane only (0.00286 ± 0.000077), suggesting that there are more inhibition of [^18^F]FLT uptake in the exposure of sevoflurane plus 7-NI than sevoflurane alone.

## Discussion

In the present study, the sevoflurane-induced inhibition on neural progenitor cells proliferation in the hippocampi were evaluated serially with microPET in conjunction with [^18^F]FLT over 4 weeks following the neonatal exposure. At the same time, the effect of 7-NI in modulating the sevoflurane-induced inhibition to neural progenitor cell proliferation was assessed. In comparison with the air-exposed rats, we observed that sevoflurane inhibited the uptake of the [^18^F]FLT in the hippocampus in the first 2 weeks following the exposure at PND 7; and inhibitory effect of sevoflurane exposure upon [^18^F]FLT uptake was absent by the fourth week after the exposure. The results also revealed that the co-administration of 7-NI did not provide protection against the sevoflurane-induced inhibition of [^18^F]FLT uptake in the hippocampus.

Neural progenitor cells retain self-renewal capability and can proliferate and differentiate into neurons and astrocytes at a rate that declines with age (51). The implication of neurogenesis in brain injury repair and functional restoration has been reported in ischemic stroke, brain traumatic injury, and neurodegenerative disorders (52–56). The enhanced neurogenesis in response to the injuries or via modulation by pharmaceutical agents has been regarded as a promising path of intervention to improve the prognosis.

It has been known that, at clinical relevant concentrations, anesthetic exposure induced apoptotic neurodegeneration and altered dendritic spine density. More recently, in *in vivo* studies, the neonatal exposure to anesthetic-induced inhibition on neurogenesis that correlated with the neurocognitive and memory functional decline in adult animals (9, 33). Furthermore, *in vitro* models of exposing cultured neural progenitor cells to general anesthetics have been utilized (35, 57, 58). It has been found that the exposure to anesthetics, which acted as either NMDAR antagonist (e.g., ketamine) or GABA_A_R agonists (e.g., isoflurane, sevoflurane, and propofol), induced inhibition of the proliferation of neural progenitor cells without inducing apoptosis or necrosis. In those studies, 5-bromo-2-deoxyuridine (BrdU) staining has been frequently used to label the newly generated cells by tagging the DNA for quantitative analysis. BrdU is a synthetic nucleoside that can be incorporated into the newly synthesized DNA strands during the synthesis (S) phase in dividing cells, and be detected by specific antibody. In comparison, [^18^F]FLT is a thymidine analogs labeled with radioactive fluorine-18 (half-life time = 109.8 min), which substituted 3′-hydroxyl group on the ribose (46). Following the initial steps of thymidine salvage pathway, FLT is transported into the cells by Na^+^-dependent active nucleoside transporters and selectively phosphorylated by TK_1_ to FLT-monophosphate or FLT-diphosphate and FLT-triphosphate (59). However, phosphorylated FLT cannot be further incorporated into DNA due to the replacement of 3′-hydroxyl group by fluorine-18, and then trapped in the cytosol (60). TK_1_ activity is absent in quiescent cells but increased in the S-phase in proliferating cells (61). The initial phosphorylation of FLT by TK_1_ is the rate-limiting step in regard of FLT intracellular retention. Therefore, TK_1_ activity determined the intracellular retention of [^18^F]FLT, which provides the basis of PET [^18^F]FLT imaging of cell proliferation. Compared with the BrdU staining, PET in conjunction with [^18^F]FLT could be performed with minimal invasiveness and could be repeated in the same subject.

In our study, the [^18^F]FLT uptake in the hippocampi were semi-quantitatively measured using the SUV derived from the static PET images acquired within 90 min. The average SUVs declined generally in week 4 in comparison with week 1, which may conform to the general activities of neural progenitor cell proliferation. The low uptake of [^18^F]FLT might be related to the limited entry of FLT through the intact brain–blood barrier and the possibility that only a fraction of progenitor cells were in the S-phase at the time of study.

The general anesthetics modulated the neural progenitor cell proliferation in an age-dependent manner, as shown in previous studies (9, 32). The anesthetic-induced inhibition to neural progenitor cells proliferation in the hippocampus had been demonstrated in 7-day-old rats (33), but not in 3- or 12-month-old rats (62). In our study, the findings about sevoflurane-induced inhibition on hippocampal [^18^F]FLT uptake were consistent with those previous studies where general anesthetics induced attenuation in the neural progenitor cell proliferation in neonatal rats (9, 32). In our study, the air-exposed rats have been taken care of by their dams during the 9-h exposure, while the sevoflurane-exposed rats were being under anesthesia without care by their dams. The asymmetry in terms of maternal care between the sevoflurane-exposed rats and the air-exposed rats might, to some extent, contribute to the difference in SUVs between them.

The mechanisms underlying general anesthetic-induced developmental neurotoxicity are recognized to be complex because multiple pathways are involved and the vulnerabilities of brain cells in different regions are disparate (35, 38, 63). Both the differentiated neurons and the neural progenitor cells could be affected by anesthetic exposure, but by different mechanisms. For example, the findings in one recent study by our group suggested that the GABA_A_R-mediated excitatory toxicity was unlikely to be implicated in the propofol-induced neural progenitor cell proliferation for functional GABA_A_Rs were not expressed in the progenitor cells (64). On the other hand, multiple pathways associated with neural progenitor cell proliferation could be involved in anesthetic exposure (35, 38). From the view of bioenergetics, the exposure to volatile anesthetics would impair the mitochondrial respiration, resulted in the loss of intracellular calcium homeostasis, production of reactive oxygen species (ROS), and compromise in adenosine triphosphate (ATP) synthesis (65). In one of our recent study, it was found that exposure of embryonic neural stem cells to propofol-induced increase in ROS production, which was attenuated by acetyl-l-carnitine that facilitates the β-oxidation of long-chain fatty acids in mitochondria (64). The free radical nitric oxide, one component of ROS, is produced in the oxidation of l-arginine to l-citrulline catalyzed by NOS. Nitric oxide (NO) is a short-life diffusible gas that has been suggested to negatively modulate neurogenesis in an autocrine or paracrine manner (30, 31). nNOS is the principal isoform expressed in the CNS. As 7-NI selectively inhibits the nNOS activity, it was expected that selective inhibition of nNOS by 7-NI and the subsequent attenuation of NO production would enhance the level of neurogenesis. However, the results showed that, in the setting of anesthetic exposure, selective inhibition of nNOS further attenuated [^18^F]FLT uptake in the hippocampi, suggesting, furthermore, inhibition of cell proliferation. In previous studies, 7-NI had been found to be protective against ketamine-induced neuronal apoptosis in forebrain neuronal culture (48). In the current study, 7-NI was found to provide no protection against the sevoflurane-induced inhibition upon neural progenitor cell proliferation. This would suggest that the disparate pathways implicated in the anesthetic neurotoxicity in differentiated neurons versus their progenitor cells. And further studies are needed to investigate the diverse functions of NO in modulation of neural progenitor cell expansion following anesthetic exposure. Collectively, the findings in our study suggested that microPET using radiolabeled tracer as cell proliferation marker, including [^18^F]FLT, detected the sevoflurane-induced attenuation of neural progenitor cell proliferation *in vivo*. microPET in conjunction with radiolabeled tracer may represent a non-invasive method to monitor the general anesthetic-induced neurotoxicity to neural progenitor cells and effects of treatment with pharmaceutical agents.

## Conflict of Interest Statement

The views in this article are based on the study presented and do not necessarily reflect any current or future opinion or position of the U.S. Food and Drug Administration. Any mention of commercial products is for clarification and not intended as endorsement. The authors declare that the research was conducted in the absence of any commercial or financial relationships that could be construed as a potential conflict of interest.
